# L-Borneol 7-O-[*β*-D-Apiofuranosyl-(1→6)]-*β*-D-Glucopyranoside Alleviates Myocardial Ischemia-Reperfusion Injury in Rats and Hypoxic/Reoxygenated Injured Myocardial Cells via Regulating the PI3K/AKT/mTOR Signaling Pathway

**DOI:** 10.1155/2022/5758303

**Published:** 2022-05-12

**Authors:** Ziyi Tong, Gaowen Li, Chengxiao Su, Liyan Zhou, Ling Zhang, Qun Chen, Qing Xia

**Affiliations:** Department of Pharmacology, Ningbo College of Health Sciences, Ningbo, Zhejiang, China

## Abstract

Ischemia/reperfusion (I/R) is a primary cause of morbidity and mortality in acute myocardial infarction (AMI). L-Borneol 7-O-[*β*-D-apiofuranosyl-(1→6)]-*β*-D-glucopyranoside (LBAG), extracted from the Radix Ophiopogonis, is the main bioactive component that may be exerting cardiovascular protection in AMI. The purpose was to examine the effects of LBAG on myocardial I/R injury (MIRI) in rats and H9c2 cells treated with hypoxia/reoxygenation (H/R). MIRI was induced through the combination of ischemia with reperfusion for 30 min and 24 h, respectively. LBAG was administered 7 days before vascular ligation. Myocardial function was detected by an electrocardiograph, histological, TTC, and TUNEL staining analyses. The influences of LBAG on the content concentration of cardiac enzymes in the serum were measured by ELISA. Moreover, H9c2 cells were exposed to LBAG or combined with AKT inhibitor (perifosine) and then exposed to H/R for simulating the cardiac injury process. Afterward, cell viability, LDH, CD-KM release, apoptosis, and autophagy were evaluated by CCK-8 and ELISA assays, flow cytometry, TUNEL, and immunofluorescence staining, respectively. Additionally, the proteins of apoptosis, autophagy, and PI3K/mTOR pathway were determined by western blotting. In I/R rats, LBAG pretreatment significantly ameliorated cardiac function, as illustrated by reducing the infarct size, myocardial autophagy, and apoptosis levels. In H/R-induced H9c2 cells, LBAG pretreatment significantly decreased cell apoptosis, LC3 II/I, and Beclin 1 levels, elevated the Bcl-2 levels, attenuated LDH, and CD-KM production. Moreover, LBAG pretreatment markedly increased the PI3K/mTOR pathway activation, and the protective influences of LBAG were partly abolished with the AKT inhibitor perifosine treatment. These findings demonstrated the protective functions of LBAG on I/R by regulating apoptosis and autophagy in vitro and in vivo by activating the PI3K/mTOR pathway.

## 1. Introduction

Currently, the high morbidity and mortality of acute myocardial infarction (AMI) ultimately lead to a heavy healthy and economic burden on individuals and society [1, 2]. The most appropriate clinical strategy to salvage ischemic heart disease is timely and effectively conducted reperfusion for restoring the blood flow in the ischemic myocardium [3, 4]. Unfortunately, reperfusion does not only rescue ischemic MI but also may evoke irreversible impairment in the myocardium, recognized as the MIRI [5, 6]. It was reported that the pathogenic factor of MIRI is related to the elevation of reactive oxygen species levels, a decrease in adenosine triphosphate production, calcium overload, suppression of mitochondrial membrane potential [5–7], apoptosis, inflammation [8], and autophagy [9]. All of these cardiovascular dysfunctions can provoke cell death and tissue damage which in turn further increase infarct size. To date, MIRI currently has been considered a major biological event depressing the effect of clinical treatment of cardiovascular diseases [10]. Although strenuous efforts had been made to develop treatment strategies for MIRI, exploring a promising drug and clarifying the therapeutic mechanisms of MIRI are urgently needed.

Apoptosis, autophagy, necroptosis, and pyroptosis processes belong to programmed cell death (PCD), which contributes to ensuring a homeostatic balance in the cells [11]. Among them, myocardial apoptosis has been confirmed as a key pathological event in MIRI [12, 13], characterized by the production of apoptotic bodies [9, 14]. Duration of I/R injury, myocardial apoptosis results in reparative fibrosis, compensatory hypertrophy, and myocardial contractile dysfunction, which ultimately aggravates myocardial injury into heart failure [15]. Autophagy, an evolutionarily ancient process, is activated to maintain the cellular, tissue, and organismal homeostasis in human diseases [16]. Many studies pointed out that basal autophagy contributes to normal cardiac homeostasis and thereby inhibits cell apoptosis in the infarct area, whereas, the excessive autophagic activity would probably cause an aggravation in MIRI [17, 18]. Type II programmed cell death, namely, autophagy, is a phenomenon of “self-eating” in cells [19]. Even though the mechanisms differ between apoptosis and autophagy, both of them share the coincident stimulating factors [19]. Furthermore, the autophagy and apoptosis in coronary heart disease are mediated by the structural and functional interactions through the complex of Beclin 1-Bcl-2/Bcl-xL [9, 20]. During the formation of the autophagosome, Beclin 1, the mammalian homolog of Atg6, participates in the formation of the autophagosomal membrane [21]. Antiapoptotic protein B-cell lymphoma/leukemia-2 (Bcl-2) is the primary member of the Bcl-2 family in apoptosis. Also, researchers have summarized that the interaction of Beclin 1 and Bcl-2 is associated with human diseases, including MIRI [22–24]. Therefore, there is a reasonable prospect that inhibiting apoptosis and excessive autophagy would be worth trying to prevent MIRI for alleviating cardiomyocyte death and conserving cardiac function.

**Figure 1:**
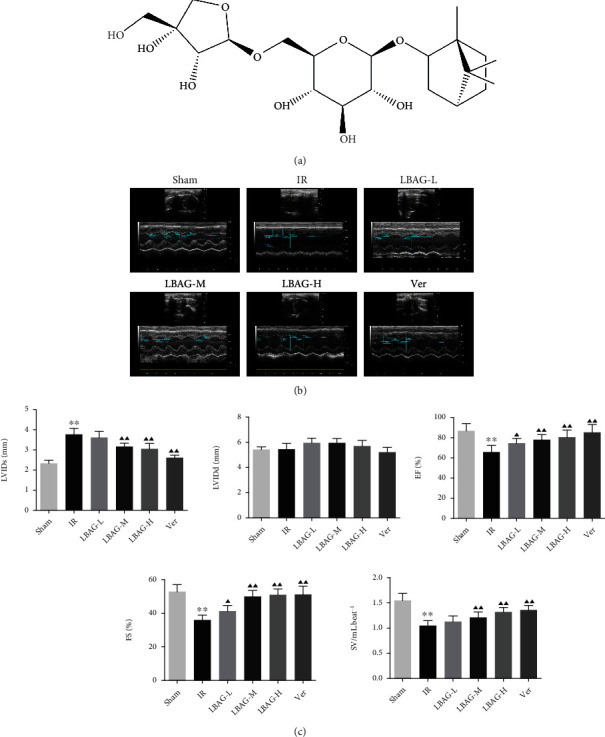
Effect of LBAG on cardiac function in rats. (a) Molecular structure of LBAG. (b) Representative M-mode images of echocardiography were performed 24 h after I/R injury in each group. (c) Statistical analysis of LVIDs, LVIDd, EF, FS, and SV. ^∗^*P* < 0.05 and ^∗∗^*P* < 0.01 vs. sham group. ^▲^*P* < 0.05 and ^▲▲^*P* < 0.01 vs. IR group.

In recent years, the cardioprotective effects of natural products and their derivatives have been drawing increasing attention from researchers in different fields. Radix Ophiopogonis, the root of Ophiopogon japonicas Ker-Gawl. (Liliaceae), is one of the most widely used Chinese herbs [25]. Previous studies demonstrated that Radix Ophiopogonis (Maidong) possesses multiple active ingredients, including glycosides, steroidal saponins, amino acids, nucleosides, and homisoflavonoids [26, 27]. Also, modern pharmacological studies have confirmed that the Methylophiopogonanone B and Ophiopogonin D have multiple physiological regulatory effects, such as immunomodulation, antioxidation, cardiovascular protection, anti-inflammation, and anticancer activities [26, 28, 29]. For example, Wang et al. reported that Ophiopogonin D attenuated heart failure by activating the Ca^2+^ homeostasis-related protein complex in H9c2 rat cardiomyoblast cells [30]. He et al. demonstrated that Methylophiopogonanone A displayed antiapoptosis activity by significantly arousing the PI3K-Akt-eNOS pathway in mice with I/R [31]. Besides this, Fan et al. have revealed that the polysaccharide extracted from the root of Radix Ophiopogonis attenuated oxidative stress on isoproterenol-induced MIRI in rats by upregulating endogenous antioxidants [32]. Additionally, L-borneol 7-O-[*β*-D-apiofuranosyl-(1→6)]-*β*-D-glucopyranoside (LBAG, C21H36O10, and C_21_H_36_O_10_; Figure 1(a)), a natural bicyclic monoterpene diglycoside compound, is the specified discrimination ingredients of Radix Ophiopogonis documented in the ZhejiangSheng ZhongYao PaoZhi GuiFan (v 2015). However, it remains unclear whether LBAG has similar protective effects of polysaccharides on MIRI. PI3K/Akt pathway is widely known to participate in cell proliferation, survival, oxidative response, metabolism, and cardiac apoptosis [33, 34]. Mammalian target of rapamycin (mTOR), a gene regulating cell growth, is positively controlled by the PI3K/Akt pathway to attenuate autophagic activity [34, 35]. For this view, we wondered if PI3K/mTOR signaling participants in the influences of LBAG on PCD.

Therefore, a rat model of MIRI and an H9c2 cell model of H/R were employed to assess the cardioprotective effects of the LBAG. In addition, the apoptosis and autophagy modulatory effects of LBAG on the PI3K/Akt/mTOR signaling cascade were investigated. Our results will help us understand the protective roles of Radix Ophiopogonis in myocardial damage and provide promising therapeutic avenues for AMI.

## 2. Materials and Methods

### 2.1. Animals and Administration

Thirty-six male rats weighing 200 ± 10 g were purchased from the Shanghai SLAC Laboratory Animal Co., Ltd. (Shanghai, China), license number: SCXK (Hu) 2017-0005. The rats were kept in an SPF environment with a 12 h-light/dark cycle at 22-24°C. After acclimatization, the rats were randomly divided into the following groups (*n* = 6): the sham group, the IR group, low-dose LBAG (LBAG-L) group, medium-dose LBAG (LBAG-M) group, high-dose LBAG (LBAG-H) group, and Verapamil (Ver) group. The animals in the LBAG-L, LBAG-M, and LBAG-H groups were given 2.5, 5, and 10 mg/kg LBAG (Yilin Bio-tech Co., Ltd.; Shanghai, China), respectively. Rats in the Ver group were given Verapamil with a dosage of 0.02 g/kg for 7 days. Besides, the sham and the IR groups were both treated with equivoluminal ultrapure water. For the last administration of the LBAG for 30 min on the seventh day, myocardial infarction operation was performed. The animal care and experimental procedures were approved by the Animal Experimentation Ethics Committee of Zhejiang Eyong Pharmaceutical Research and Development Center (No. SYXK (Zhe)2021-0033, Zhejiang, China).

### 2.2. MIRI in Rats

The rats were anesthetized before the myocardial infarction operation. A 6-0 silk thread was chosen to tie the left anterior descending coronary artery for 30 min ischemia. Electrocardiogram showed ST-segment elevation and a color change of the myocardial tissue, indicating successful ischemia. Subsequently, the reperfusion was performed for 2 h. The animals in the sham group treated the coincident procedure without ligation. After the operation for 24 h, the cardiac function of rats was estimated with echocardiography. Next, the blood was taken from the abdominal aorta of each rat and then centrifuged to obtain the serum for biochemical analysis.

### 2.3. Echocardiography

After MIRI, the rats were anesthetized with 1-2% isoflurane. Then, the animal ultrasound imaging system (Vevo 1100, VisualSonic, Canada) was employed to assess cardiac function. The M-mode images were collected by MS250 ultrasound probe. The parameters of the left ventricular end-diastolic diameter (LVIDd), left ventricular internal dimension systole (LVIDs), ejection fraction (EF), fractional shortening (FS), and stroke volume (SV) were recorded and calculated. After that, all animals were euthanized, and then, the myocardial tissues were quickly removed for further analyses.

### 2.4. Determination of Serum CK-MB, cTnT, AST, LDH, CK, and LDH-1 in Rats

Serum creatine kinase isoenzyme (CK-MB), cardiac troponin T (cTnT), lactic dehydrogenase (LDH), aspartate transaminase (AST), creatine kinase (CK), and lactate dehydrogenase isoenzyme 1 (LDH-1) contents in I/R injury rats can indirectly reflect the degree of myocardial injury. Then, the abovementioned myocardial injury markers were measured using commercial kits.

### 2.5. HE (Hematoxylin-Eosin) and Triphenyltetrazolium Chloride (TTC) Staining

Myocardial tissues were immersed in 4% paraformaldehyde for 3 days. After embedded, the embedded heart tissue specimens were cut into 4 *μ*m thick sections, and then, the sections were stained with HE. Additionally, the infarct sizes of the myocardium were estimated by TTC staining. Under microscopic observation, the infarct area was identified by the white color area and the viable tissue was recognized as the red coloration. At last, the Image-Pro Plus 6.0 software (Wokingham, UK) was employed to assess infarct size.

### 2.6. TUNEL Assay

The left ventricular tissues and H9C2 cell apoptosis were assessed with TUNEL assay (Millipore, USA). Briefly, the paraffin-embedded tissues were sectioned on slides at 4 *μ*m thickness. After counter-staining with hematoxylin, the sections were observed with an optical microscope (Nikon Corporation, Japan). According to the manufacturer's protocol, the apoptosis of LBAG-treated H9c2 cells was detected using the One Step TUNEL Apoptosis Assay Kit (Beyotime, China). After TUNEL staining, the nuclear was stained using 5 *μ*g/mL DAPI (Beyotime, China). The apoptosis-positive cells were stained brown-yellow and the normal nucleus blue. Five randomly selected fields of view were examined, and the apoptotic cells of each view were counted under an inverted fluorescence microscope. At last, the results were presented as a percentage (%) of apoptotic cells using the following formula: the percentage of TUNEL positive cells (%) = (apoptotic nuclei count/total nucleus count) × 100%.

### 2.7. Cell Culture

Rat embryonic ventricular myocyte H9c2 cells were obtained from the Shanghai Institute of Biochemistry and Cell Biology (Shanghai, China) and cultured in DMEM medium (Invitrogen, USA) supplemented with 10% of FBS (Invitrogen, USA) under 95% air, 5% CO_2_ at 37°C.

### 2.8. H/R Model and LBAG Administration

The H/R model of H9c2 cells was established to mimic myocardial injury in vivo. Briefly, H9c2 cells (1 × 10^5^ cell/well) were placed in six-well plates and randomly divided into the following six groups: (1) control group, (2) H/R group, (3) 0.1 *μ*mol/L LBAG group, (4) 1 *μ*mol/L LBAG group, (5) 10 *μ*mol/L LBAG group, and (6) 10 *μ*mol/L LBAG+5 *μ*mol/L perifosine (Per, Viva Bioscience, England). The H/R injury was mediated by putting H9c2 cells into hypoxic surroundings (95% N_2_ and 5% CO_2_) for 6 h. Next, reoxygenation was performed by placing H9c2 in the normal culture environments for an additional 6 h. H9c2 cells in the control group were cultured under normoxic conditions. LBAG with concentrations of 0.1, 1, and 10 *μ*mol/L was mixed with the fresh Dulbecco's Modified Eagle Medium (DMEM) for 24 h before H/R injury, and the cellular morphology of H9c2 cells was observed using the optical microscope. Additionally, the LDH and CK-MB content levels in the supernatant were detected by the ELISA assay.

### 2.9. CCK-8 Assay

H9c2 cell viability was assessed using the CCK-8 assay (Sigma, USA). Briefly, the H9c2 cells (2 × 10^3^ cells/well) were placed in 96-well plates for 24 h. Then, cells were pretreated with LBAG or combined perifosine followed by H/R treatment. Afterward, 10 *μ*l CCK-8 solutions were put into 96-well plates and incubated at 37°C for an additional 2 h. The optical density was recorded under 450 nm wavelength by a microplate reader (Thermo Scientific).

### 2.10. Apoptosis Assay

After H9c2 cells had been treated with the LBAG and H/R, H9c2 cells' apoptosis was determined by using a commercial cell apoptosis kit (Solarbio, Beijing, China). The apoptosis rate was calculated according to the upper and lower right quadrants of the plots for each group, respectively, using the FlowJo X software (Tree Star, Inc.).

### 2.11. Immunofluorescence Assay

Immunofluorescence analysis of H9c2 cells with LBAG treatment was employed to assess the levels of autophagy-related protein, p62. In short, the H9c2 cells were fixed, washed, and then incubated with the diluted p62 antibody (1 : 200, AF5384, Affinity Biosciences Ltd., USA) at 4°C overnight. Ultimately, the images were captured using a microscope.

### 2.12. Western Blot Assay

Total protein of the left ventricular tissue of rats and H9c2 cells with LBAG treatment was extracted using lysis buffer containing PMSF on ice. The protein levels were estimated with a BCA method (Thermo Fisher Scientific). Equivalent protein (20 *μ*g) was separated by 10% SDS-PAGE and transferred to PVDF membranes (Millipore, USA). After being blocked, the membranes were then incubated with primary antibodies including Bax (1 : 2000, AF0120, Affinity), Bcl-2 (1 : 1000, AF6139, Affinity), cleaved-caspase 3 (1 : 1000, AF7022, Affinity), p62 (1 : 2000, AF5384, Affinity), LC3 (1 : 1000, AF5402, Affinity), Beclin 1 (1 : 1000, AF5128, Affinity), PI3K (1 : 1000, AF6241, Affinity), phospho-PI3K (1 : 1000, AF3241, Affinity), AKT (1 : 1000, AF6264, Affinity), phospho-pan-AKT1/2/3 (Ser473) (1 : 1000, AF0016, Affinity), mTOR (1 : 2000, AF6308, Affinity), phospho-mTOR (Ser2448) (1 : 1000, AF3308, Affinity), and *β*-actin (1 : 5000, AF7018, Affinity) overnight at 4°C. Subsequently, the membranes were incubated with secondary antibodies (1 : 5000, Kangchen, China) for 1.5 h at room temperature. Protein bands were visualized using an enhanced chemiluminescent (ECL) kit (Beyotime, China). The image intensity was analyzed using the Image J software.

### 2.13. Statistical Analysis

All data were expressed as the mean ± standard deviations. Statistical differences between the groups were performed using a one-way ANOVA. *P* < 0.05 was considered statistically significant.

## 3. Results

### 3.1. LBAG Ameliorated Cardiac Function of MIRI Rats

**Figure 2:**
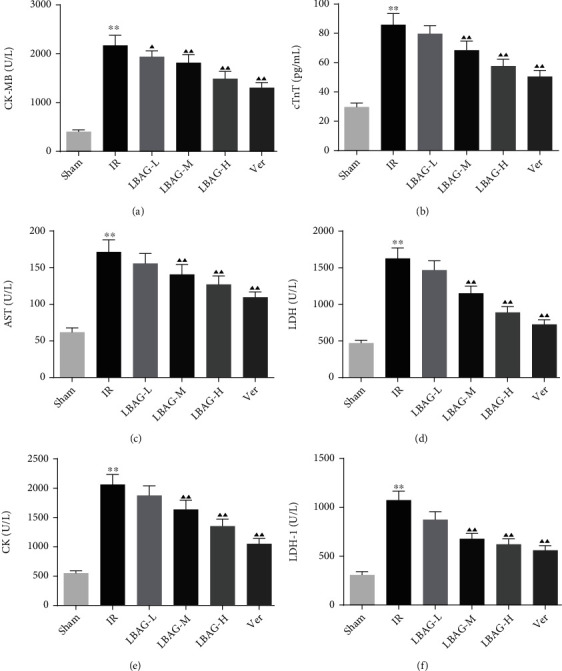
Effect of LBAG on cardiac damage markers in rats. The level of (a) CK-MB, (b) cTnT, (c) AST, (d) LDH, (e) CK, and (f) LDH-1 in the serum of rats with myocardial injury after LBAG treatment. ^∗^*P* < 0.05 and ^∗∗^*P* < 0.01 vs. sham group. ^▲^*P* < 0.05 and ^▲▲^*P* < 0.01 vs. IR group.

**Figure 3:**
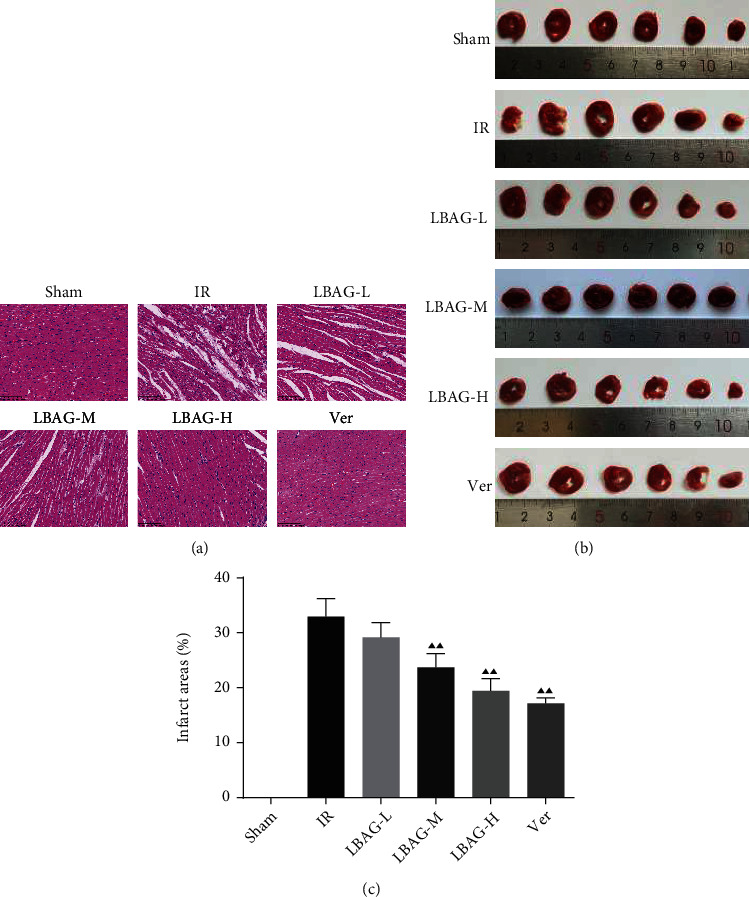
Effect of LBAG on pathological features of heart in rats. (a) The histopathological changes of the heart in LBAG-treated I/R-injured rats were measured through HE staining (scale bar, 100 *μ*m). (b) Representative TTC-stained images of the heart in LBAG-treated I/R-injured rats. (c) Quantitative analysis of myocardial infarction area. ^▲^*P* < 0.05 and ^▲▲^*P* < 0.01 vs. IR group.

The results of echocardiography showed that the value of LVIDs in the IR group was significantly upregulated than that in the sham group, accompanied by an obvious downregulation of EF, FS, and SV, pointing out that cardiac function and structure were badly damaged after MIRI; however, we found that there was no difference of the value of LVIDd among each group in this study (Figures 1(b) and 1(c)). With LBAG and Ver pretreatment, the LVIDs was significantly attenuated, in addition to increased EF, FS, and SV were found in the LBAG and Ver groups. Furthermore, the ELISA assay results found that the content levels of the MIRI markers CK-MB, cTnT, AST, LDH, CK, and LDH-1 were significantly increased in the IR group (Figures 2(a)–2(f)). Interestingly, administration of LBAG or Ver significantly decreased the CK-MB, cTnT, AST, LDH, CK, and LDH-1 levels compared to that in the IR group. For cardiac histopathological analysis, as illustrated in Figure 3(a), the HE staining showed that the myocardial cells were distinctly irregularly arranged and partially lysis in the infarct border zone, as well as breakage of cardiac muscle fibers, was observed in tissue sections from the IR group, which was partially reversed by LBAG or Ver pretreatment. To further examine the role of LBAG, a TTC staining assay was selected to quantify the infarct area in rat hearts (Figure 3(b)). Myocardial infarction size was increased in MIRI rats. Besides, pretreatment with LBAG or Ver significantly decreased the infarct areas, compared with the IR group (Figure 3(c)). Taken together, these results suggested that pretreatment with LBAG contributes to against myocardial damage during I/R in rats.

### 3.2. LBAG Inhibited Cardiomyocyte Apoptosis and Autophagy in MIRI Rats

**Figure 4:**
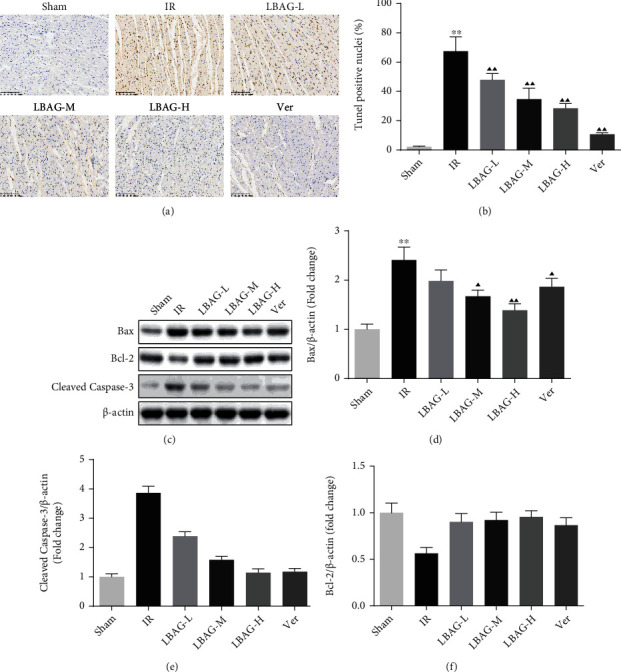
Effect of LBAG on cardiomyocyte apoptosis in rats. (a) Cardiac apoptosis was assessed by TUNEL staining (magnification, ×200). (b) Positive apoptosis cells in each group. (c) Western blot analysis and statistical analysis of (d) Bax, (e) cleaved caspase-3, and (f) Bcl-2 proteins in each group. ^∗^*P* < 0.05 and ^∗∗^*P* < 0.01 vs. sham group. ^▲^*P* < 0.05 and ^▲▲^*P* < 0.01 vs. IR group.

**Figure 5:**
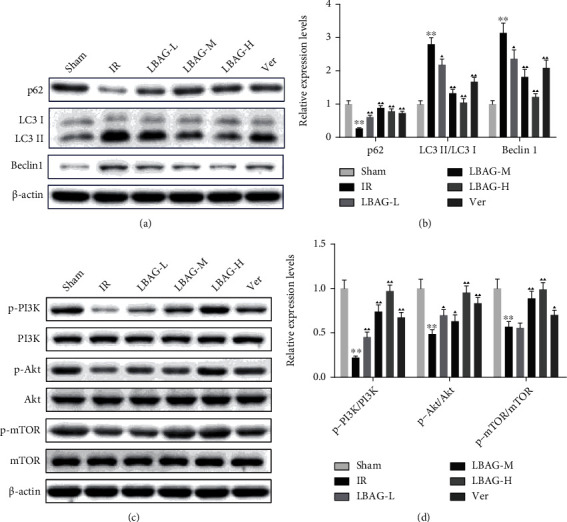
Effect of LBAG on the expression of autophagy and PI3K/AKT pathway-related proteins in rats. (a) Representative protein blots of p62, LC3, and Beclin 1. (b) Quantitative analysis of p62, LC3, and Beclin 1 proteins. (c) Representative protein blots of PI3K/AKT pathway-related proteins. (d) Quantitative analysis of PI3K pathway-related targets in each group. ^∗^*P* < 0.05 and ^∗∗^*P* < 0.01 vs. sham group. ^▲^*P* < 0.05 and ^▲▲^*P* < 0.01 vs. IR group.

As shown in Figures 4(a) and 4(b), TUNEL staining illustrated that the apoptotic cells were apparently higher in the IR group, while this increase was attenuated by LBAG pretreatment. Besides, the Bcl-2, Bax, and caspase-3 levels were measured with western blotting. Notably, we discovered that the cleaved caspase-3 and Bax levels were observably upregulated, and the Bcl-2 level was significantly decreased in the IR group (Figure 4(c)). Compared to that in the I/R group, the Bax and cleaved caspase-3 levels were significantly reduced (Figures 4(d) and 4(e)), and the Bcl-2 was upregulated in the LBAG groups (Figure 4(f)). To identify whether LBAG inhibits autophagic flux in MIRI rats, the levels of p62, LC3, and Beclin 1 were measured by western blot. Western blotting showed that the level of the p62 protein was higher in LBAG groups, while the level of the LC3 and Beclin 1 was lower in LBAG groups than in the IR group (Figures 5(a) and 5(b)). As we know, the PI3K/mTOR pathway is closely involved in autophagy in the disease process [36]. Based on this, the levels of PI3K/mTOR pathway-related targets were also estimated with western blot assay. Western blot results suggested that the phosphorylation of PI3K, AKT, and mTOR protein levels in the myocardium of the LBAG groups was upregulated significantly compared with the IR group (Figures 5(c) and 5(d)). These results suggested that LBAG might restrict MIRI-induced apoptosis and autophagy activation by activating PI3K/mTOR pathway in the rat heart.

### 3.3. LBAG Enhanced H9c2 cell Viability during H/R

**Figure 6:**
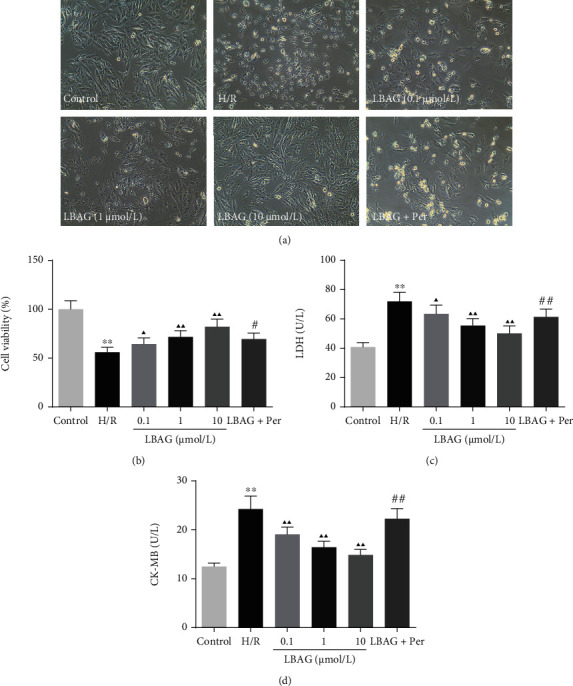
Effect of LBAG on the cell injury of H/R model in H9c2 cells. (a) Cell morphology of H/R-induced H9c2 cells treated with LBAG was photoed by optical microscopy. (b) Cell viability of the H/R injury model treated with LBAG was assessed by CCK-8 assay. (c, d) The LDH and CK-MB levels in the culture supernatant of H/R-injured H9c2 cells were detected using ELISA. ^∗^*P* < 0.05 and ^∗∗^*P* < 0.01 vs. control group. ^▲^*P* < 0.05 and ^▲▲^*P* < 0.01 vs. H/R group. ^#^*P* < 0.05 and ^##^*P* < 0.01 vs. 10 *μ*mol/L LBAG group.

The morphological investigation of H/R-mediated H9c2 cells with LBAG treatment was performed by fluorescence inverted microscope. As shown in Figure 6(a), we can see that cell lysis and vacuolar changes of H9c2 cells are much stronger than that of control cells. With LBAG treatment of different doses, the prism structures and close arrangement of H9c2 cells in the control and LBAG groups can be observed compared with the H/R group. Moreover, the cell lysis and vacuolar changes of H9c2 cells in the 10 *μ*mol/L LBAG+Per group are much heightened than that of the 10 *μ*mol/L LBAG group. Besides, the CCK-8 assay was chosen to investigate the effect of LBAG on the cell viability of H/R-mediated H9c2 cells. These results indicated that the LBAG increased proliferative capacity of the H/R-mediated H9c2 cells (Figure 6(b)). And we then further investigated the LDH and CK-MB levels in the culture supernatant of the myocardial H9c2 cell line. As shown in Figures 6(c) and 6(d), LDH and CK-MB content levels in LBAG-treated groups were much lower than that compared with the H/R group. Besides, we found that perifosine, as an AKT inhibitor, contributed to reversing the effects of LBAG on cell viability, LDH, and CK-MB content levels in the H/R-induced H9c2 cells.

### 3.4. Inhibition of AKT Weakens the Effects of LBAG Treatment in H/R-Induced H9c2 Cells

**Figure 7:**
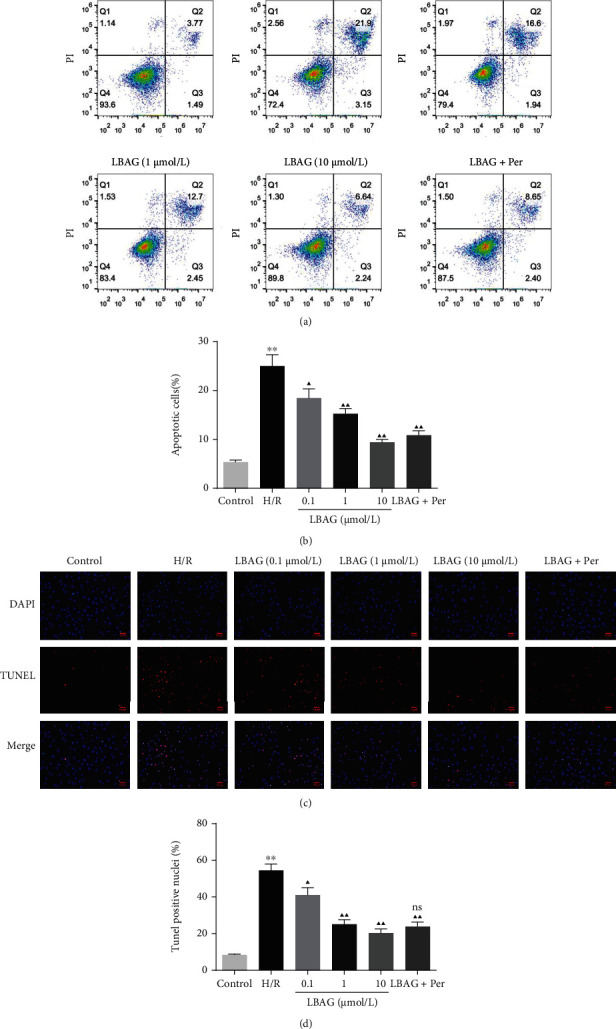
Effect of LBAG on the cell apoptosis of H/R-injured H9c2 cells. (a, b) Representative photographs of flow cytometry using FITC/PI double staining in H/R-injured H9c2 cells treated with LBAG. (c, d) Representative photographs of TUNEL assay in LBAG-treated H/R-injured H9c2 cells. Apoptosis index significantly decreased following LBAG treatment in H/R-injured cardiomyocytes. ^∗^*P* < 0.05 and ^∗∗^*P* < 0.01 vs. control group. ^▲^*P* < 0.05 and ^▲▲^*P* < 0.01 vs. H/R group. No significance (ns) vs. 10 *μ*mol/L LBAG group.

**Figure 8:**
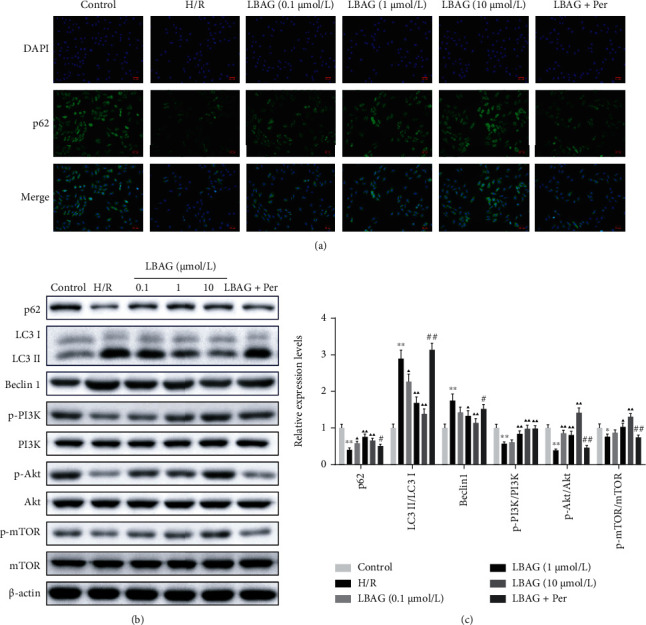
Effect of LBAG on the expression of autophagy and PI3K/AKT pathway-related proteins in H9c2 cells with H/R injury. (a) p62 expression was detected by immunofluorescence analysis in LBAG-treated H/R-injured H9c2 cells. Scale bar: 50 *μ*m. (b, c) p62, LC3, Beclin 1, and PI3K/AKT pathway-related protein levels were estimated with western blotting in LBAG-treated H/R-injured H9c2 cells. ^∗^*P* < 0.05 and ^∗∗^*P* < 0.01 vs. control group. ^▲^*P* < 0.05 and ^▲▲^*P* < 0.01 vs. H/R group. ^#^*P* < 0.05 and ^##^*P* < 0.01 vs. 10 *μ*mol/L LBAG group.

Immediately afterward, we wonder whether LBAG suppresses apoptosis and autophagy progression through AKT. Flow cytometry showed that the apoptotic cell number was increased in the H/R-treated group; meanwhile, the apoptosis rate was remarkably lower in LBAG-treated H/R-induced H9c2 cells (Figures 7(a) and 7(b)). Also, similar results are seen in the TUNEL assay, except for the large magnitudes (Figures 7(c) and 7(d)). To further investigate the antiautophagy effect of LBAG in vitro, the p62 expression levels were also investigated as shown in Figures 8(a) and 8(b). LBAG remarkably increased p62 expression levels; meanwhile, the levels of LC3 and Beclin 1, other autophagy-dependent markers, were also significantly reduced in H/R-mediated H9c2 cells. Moreover, the western blotting assay also revealed that p-PI3K, p-AKT, and p-mTOR protein levels were elevated in H/R-mediated H9c2 cells treated with LBAG. Additionally, AKT inhibition weakened the inhibitory influence of LBAG on apoptosis and autophagy in H/R-mediated H9c2 cells. Compared with the 10 *μ*mol/L LBAG group, further investigations revealed that the decreased AKT observably upregulated the LC 3 and Beclin 1 protein levels and decreased p62 and p-mTOR expressions in H/R-mediated H9c2 cells (Figures 8(b) and 8(c)). Thus, these data indicated that LBAG ameliorates H/R progression through the PI3K-AKT-mTOR pathway.

## 4. Discussion

In this study, we studied the influences of LBAG in MIRI rats and H/R-mediated injury in H9c2 cells. After 1 week of LBAG pretreatment, the myocardial function of rats was measured by echocardiography. We illustrated that there was significant difference in LVIDs, EF, FS, and SV among the IR model and LBAG groups. Furthermore, the value of LVIDs was lower in the rats of LBAG groups, and the EF, FS, and SV of the left ventricle in the rats of LBAG groups were also higher than that in the IR group. Besides, our ELISA results found that the levels of CK-MB, cTnT, AST, LDH, CK, and LDH-1 in serum were observably increased after 24 h of reperfusion, which is similar to the study of Yao et al. [37]. The results showed that LBAG treatment improved the cytokine release following MI/R in rats. The following H&E, TTC, and TUNEL staining indicated that LBAG ameliorated cardiac dysfunction and exerted antiapoptotic and antiautophagic effects in vivo. Besides, myocardial cell injury is related to apoptosis and autophagy activation, evidenced by a decrease in p62 levels and an increase in the LC3 and Beclin1 levels in vivo and in vitro. Also, LBAG could affect the autophagy-related proteins via the PI3K/AKT/mTOR signaling. All results suggested that LBAG regulated the process of apoptosis and further alleviated the autophagy in myocardial cell injury.

In this study, the MIRI changes in myocardial ultrastructure and infarction size in rats. It has previously been demonstrated that the damage of myocardial structure induces the increased release of CK-MB, cTnT, AST, LDH, CK, and LDH-1 from myocardial tissues [38, 39]. These enzymes as heart-specific biochemicals have proven valuable in the diagnosis of myocardial damage [40, 41]. The current study found that the CK-MB, cTnT, AST, LDH, CK, and LDH-1 levels in serum were increased in MIRI rats, accompanied by an increase of LDH and CK-MB levels in H/R-injured H9c2 cells and that decreased under pretreatment with LBAG or Ver. These results suggest that LBAG attenuated I/R injury-induced myocardial damage in rats.

After ischemia, reperfusion sometimes itself may cause persistent secondary myocardial injury. Accumulating evidence has demonstrated that myocardial damage was also associated with multiple critical pathogenic factors, including inflammation, oxidative stress, apoptosis, and autophagy [42]. Apoptosis, as one of the programmed cell death forms, may have been caused by IR, which affects the development of MI and the repair of myocardial injury [43]. The Bcl-2 family consists of the proapoptotic Bax and the antiapoptotic Bcl-2, and a Bcl-2/Bax ratio also was generally used to assess the antiapoptotic ability of cells under external stimulations [44]. Cleaved caspase-3 has been proved to be of importance in the apoptosis process, which is an executor of apoptosis [37, 45, 46]. Study evidence has demonstrated that apoptosis is one of the main pathogenic mechanisms of MIRI. For example, Gong et al. reported that I/R resulted in myocardial cell apoptosis through detection of cleaved-caspase 3, Bax, and TUNEL and that cotreatment of rosuvastatin with dapagliflozin synergistically inhibited apoptosis in H/R-induced H9c2 cells [47]. Similarly, treatment with LBAG significantly inhibited apoptosis, by reducing Bax and cleaved-caspase 3 expressions and alleviating the Bcl-2 levels in heart tissues of MIRI rats. Also, flow cytometry and the TUNEL assays have been used to evaluate the programmed cell death in vitro model. We confirmed that an increase of apoptotic cells following H/R damage was decreased by LBAG treatment in H9c2 cells. Therefore, the antiapoptosis effect of LBAG may be the key to alleviating myocardial damage caused by IR.

Growing studies showed that autophagy contributed to the pathological process of cardiac I/R injury [18, 20, 48]. In the process of autophagy, Beclin 1 is needed to drive autophagy vesicle nucleation, and a study by Zhang et al. showed that IR could increase Beclin 1 levels [49]. Most importantly, the autophagy-promoting protein LC3, as a marker for autophagosomes, is essential in the ensuing autophagosome elongation step [50]. Additionally, p62 is known as an autophagy adaptor molecule of the autophagic process [51]. To our knowledge, cross-talk between the Bcl-2 and Beclin 1 occurs in a wide range of physiological and pathological situations, including AMI. In the present study, we identified that LC3II/I and Beclin 1 levels were increased in heart tissues and H9c2 cells after IR or H/R, respectively, while the expression of p62 was decreased, which illustrated autophagy was initiated in AMI. Herein, our results showed that LBAG inhibited the autophagy targets, which further suggested that inhibiting autophagy and apoptosis of LBAG is an effective strategy for relieving AMI.

PI3K-Akt-mTOR pathway, a highly conserved pathway, has been shown to play important roles in cell proliferation, oxidation, inflammation, cell survival, and apoptosis. A study reported that the p-AKT level was decreased in heart tissue after IR, which suggested that the PI3K/AKT pathway was inactivated in AMI after MIRI [52]. In the current research, we also noticed that PI3K/mTOR pathway was inactivated in heart tissue and H9c2 cells after IR or H/R, respectively. More importantly, we further found that LBAG activated PI3K/mTOR pathway in IR-induced rats and H/R-induced H9C2 cells, as indicated by the increased phosphorylation levels of PI3K, AKT, and downstream effector of mTOR. Interestingly, we inhibited AKT with perifosine and observed that the AKT-mTOR signaling pathway inhibition blocked the effects of LBAG on cell viability, cell apoptosis, and autophagy of H/R-induced H9c2 cells. These results concluded that PI3K/mTOR pathway participated in the regulatory activity of LBAG on apoptosis and autophagy, and PI3K/mTOR pathway-mediated apoptosis and autophagy participated in the protective influence of LBAG on AMI induced by MIRI.

In summary, these results demonstrate that LBAG could ameliorate IR-induced cardiomyocyte injury through inhibition of myocardium apoptosis and autophagy, as well as the activation of PI3K/mTOR pathway. Moreover, LBAG pretreatment could be a promising therapeutic pattern for the intervention of AMI patients.

## Data Availability

The datasets used and/or analyzed during the current study are available from the corresponding author on reasonable request.
